# Early Intraprosthetic Dislocation After Closed Reduction of Dual-Mobility Total Hip Arthroplasty: A Case Report

**DOI:** 10.7759/cureus.103683

**Published:** 2026-02-15

**Authors:** João C Mendes, Gonçalo Fernandes, Daniela Isidoro, Catarina Corte-Real, Fernando Judas

**Affiliations:** 1 Department of Orthopedics, Unidade Local de Saúde de Coimbra, Coimbra, PRT

**Keywords:** bottle-opener effect, dual-mobility total hip arthroplasty, early intraprosthetic dislocation, hip arthroplasty instability, hip closed reduction complications, iatrogenic polyethylene liner dissociation

## Abstract

Intraprosthetic dislocation (IPD) is a rare complication of dual-mobility (DM) total hip arthroplasty (THA), defined as dissociation of the polyethylene (PE) liner from the femoral head. While IPD has classically been linked to late-stage PE wear and long-term mechanical failure, recent studies and case reports have increasingly identified early, iatrogenic IPD as a potential complication following closed reduction of dislocated DM THA components. We report the case of a 75-year-old male who underwent THA with a DM acetabular component following a femoral neck fracture. Sixteen days after surgery, the first dislocation occurred and was managed with a bedside closed reduction performed without anesthesia or fluoroscopic guidance, which showed no immediate evidence of PE liner dissociation. Three days later, a second dislocation occurred. Radiographs revealed an eccentrically positioned femoral head, and computed tomography confirmed PE liner migration. Revision surgery identified the liner in the gluteus medius muscle. A new cemented DM component was implanted; the femoral stem was retained in place. This case highlights the importance of prevention of iatrogenic IPD through appropriate use of anesthesia and fluoroscopic control during closed reductions. Early recognition remains critical to avoid further complications. Gentle manipulation under fluoroscopic guidance is essential to prevent iatrogenic damage. Radiographic signs of eccentric head positioning or absence of the liner should prompt immediate evaluation.

## Introduction

Dual-mobility (DM) implants, first introduced by Gilles Bousquet in 1974, combine a small articulation to minimize wear with a larger articulation that improves hip stability and increases jump distance [1]. Over the past decades, DM components have gained popularity in both primary and revision total hip arthroplasty (THA), particularly in patients at high risk for dislocation [2,3].

Intraprosthetic dislocation (IPD), defined as dissociation of the polyethylene (PE) liner from the femoral head, is a specific complication of DM implants. Although it was historically attributed to long-term PE wear and mid- to late-term failure [4], early iatrogenic IPD is now increasingly recognized, particularly after forceful closed reductions of DM THA [5].

We present a case of early IPD following closed reduction of a DM THA in an elderly patient with two early dislocations, highlighting the mechanisms, diagnostic challenges, and surgical management of this rare complication.​​​​​

## Case presentation

A 75-year-old man presented to the emergency department in November 2024 with acute left hip pain, inability to bear weight, and functional impairment after a low-energy fall. His body mass index was 24. Medical history included type 2 diabetes mellitus and alcohol use disorder under neurological follow-up. Before the injury, he was independently mobile without walking aids and reported no prior hip pain.

**Figure 1:**
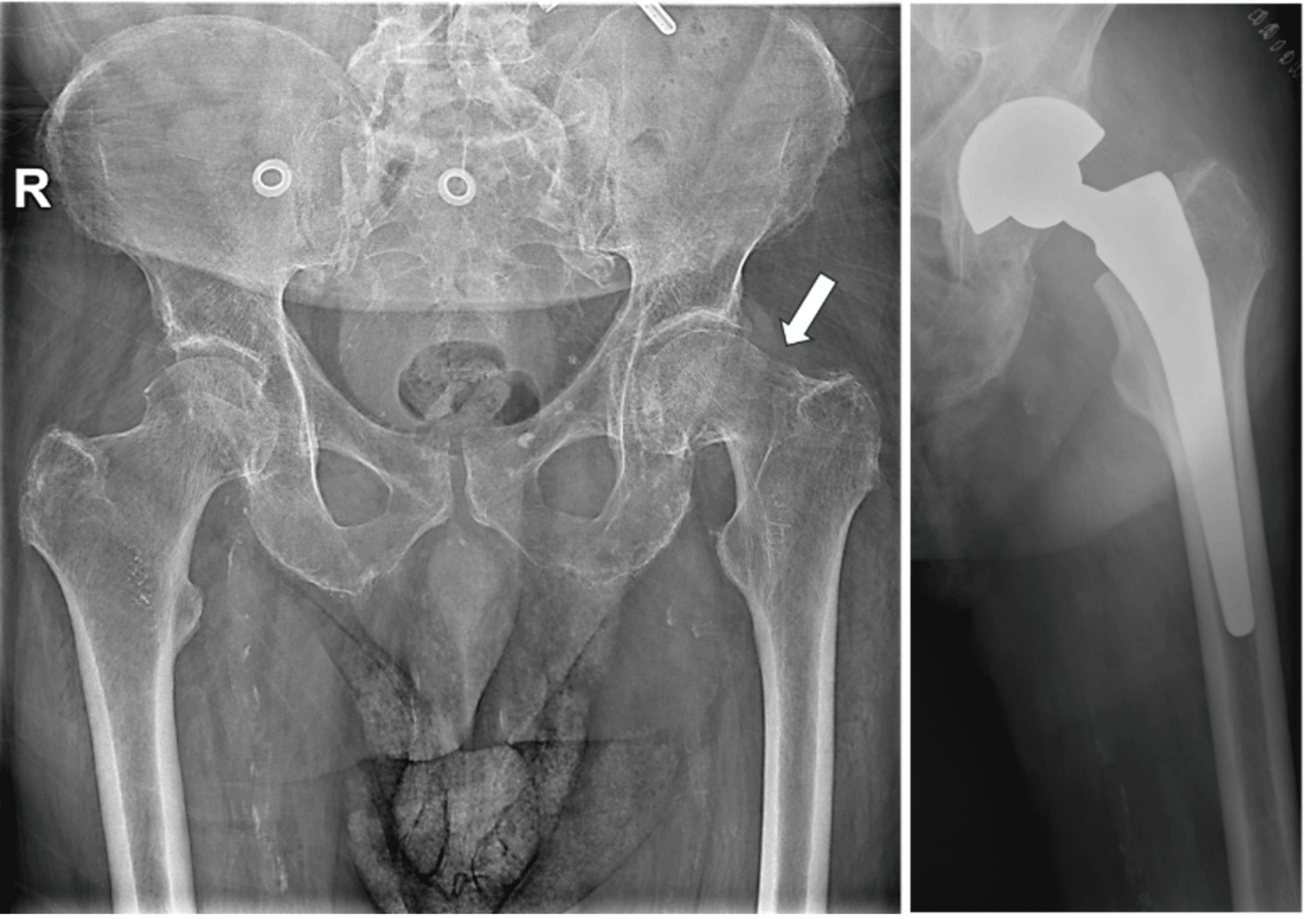
Pelvic radiograph showing displaced femoral neck fracture (arrow) treated with dual‑mobility total hip arthroplasty.

Plain radiographs demonstrated a displaced left femoral neck fracture. The patient underwent THA using a DM acetabular construct to reduce the risk of postoperative instability. A cementless G7® acetabular cup and an Avenir® cementless femoral stem (Zimmer Biomet, Warsaw, IN, USA) were implanted (Figure 1). The perioperative course and the initial five days of hospitalization were uneventful.

**Figure 2:**
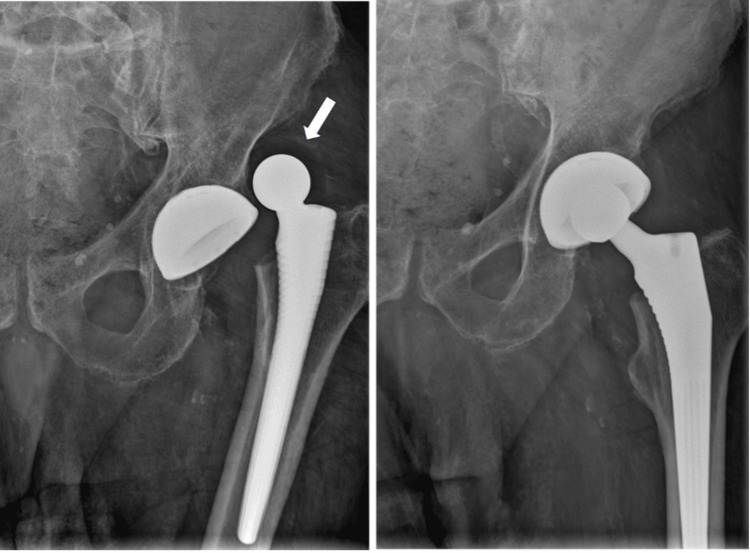
Hip radiographs obtained 16 days postoperatively demonstrating dislocation of the dual-mobility construct, with the femoral head-liner displaced from the acetabular cup (arrow).

On postoperative day 16, the patient returned with acute hip pain, limb shortening, and functional impairment after slipping in the bathroom, without reporting a direct impact to the hip. Radiographs confirmed a posterior-superior dislocation of the prosthetic hip (Figure 2). On the anteroposterior pelvis radiograph, the acetabular component showed no radiographic signs of loosening or gross malposition; inclination was estimated as within an acceptable range on plain films, while version could not be reliably quantified on the available radiographs. Closed reduction was performed in the emergency department without general anesthesia due to the urgent clinical scenario and limited immediate access to an operating room and anesthesia support; the procedure was conducted with analgesia and procedural sedation following institutional protocol. Post-reduction imaging appeared concentric. However, subtle eccentricity or early compromise of the head-liner interface may have been obscured at that time (Figure 2).

**Figure 3:**
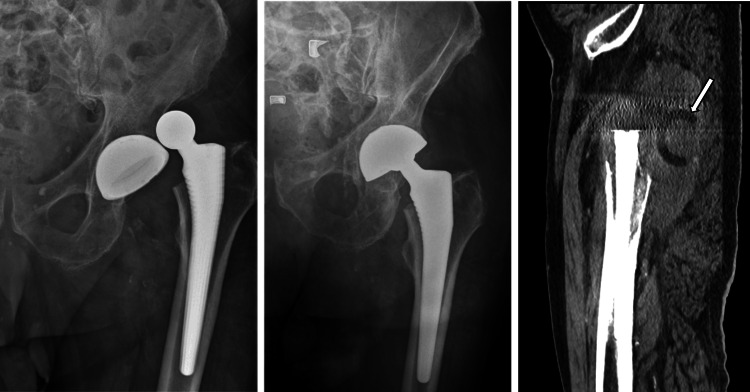
Post-reduction hip radiograph demonstrating eccentric femoral head position. Sagittal CT image confirms polyethylene liner dissociation with migration into the peritrochanteric soft tissues - “bubble sign” (arrow). CT, computed tomography.

Three days later (postoperative day 19), the patient re-presented with a second dislocation. No new major trauma was reported preceding this episode. After closed reduction, radiographs demonstrated eccentric femoral head positioning within the metal cup and absence of a clearly visualized PE liner. Computed tomography (CT) confirmed dissociation of the PE liner from the femoral head and migration toward the greater trochanter (Figure 3).

**Figure 4:**
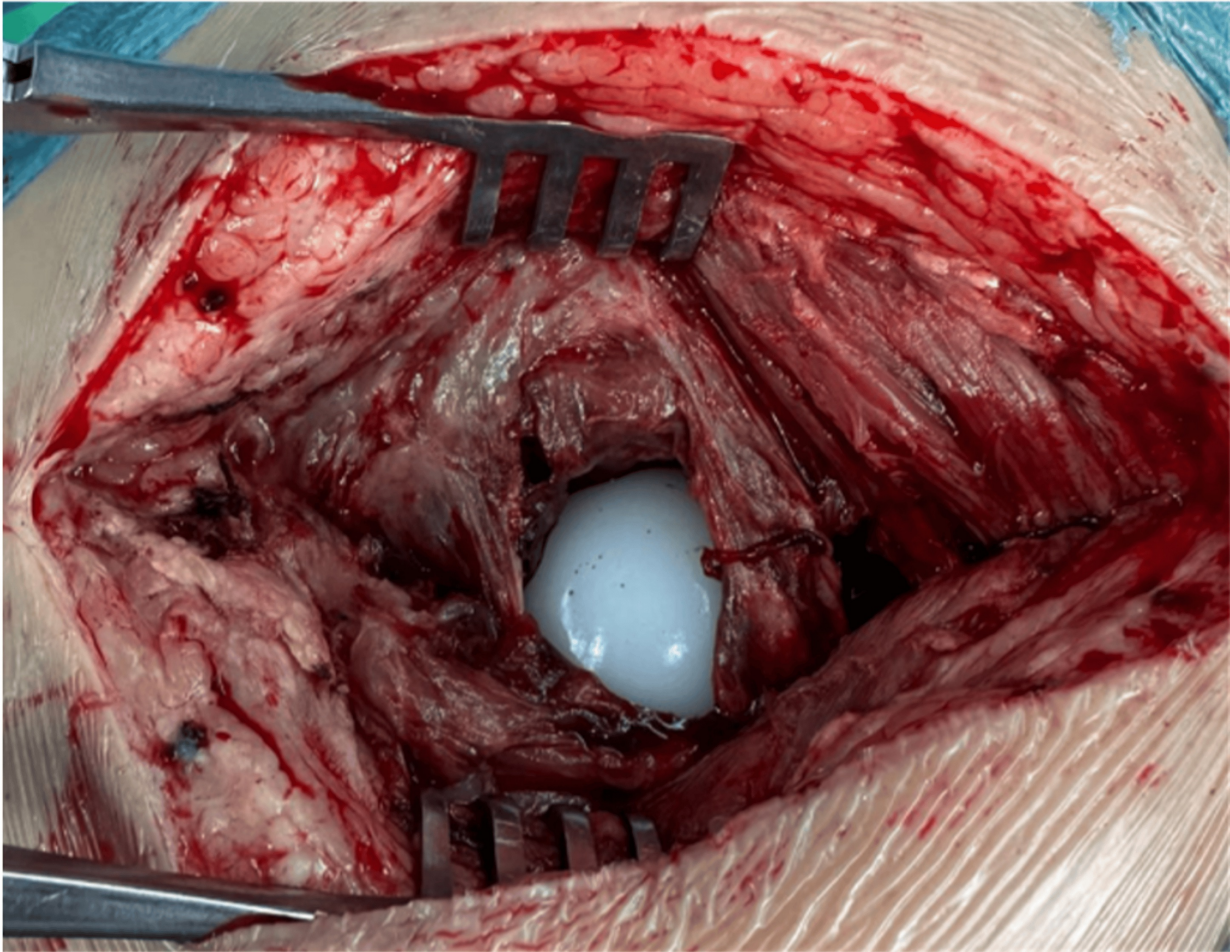
Intraoperative photograph of polyethylene liner retrieved from gluteus medius.

**Figure 5:**
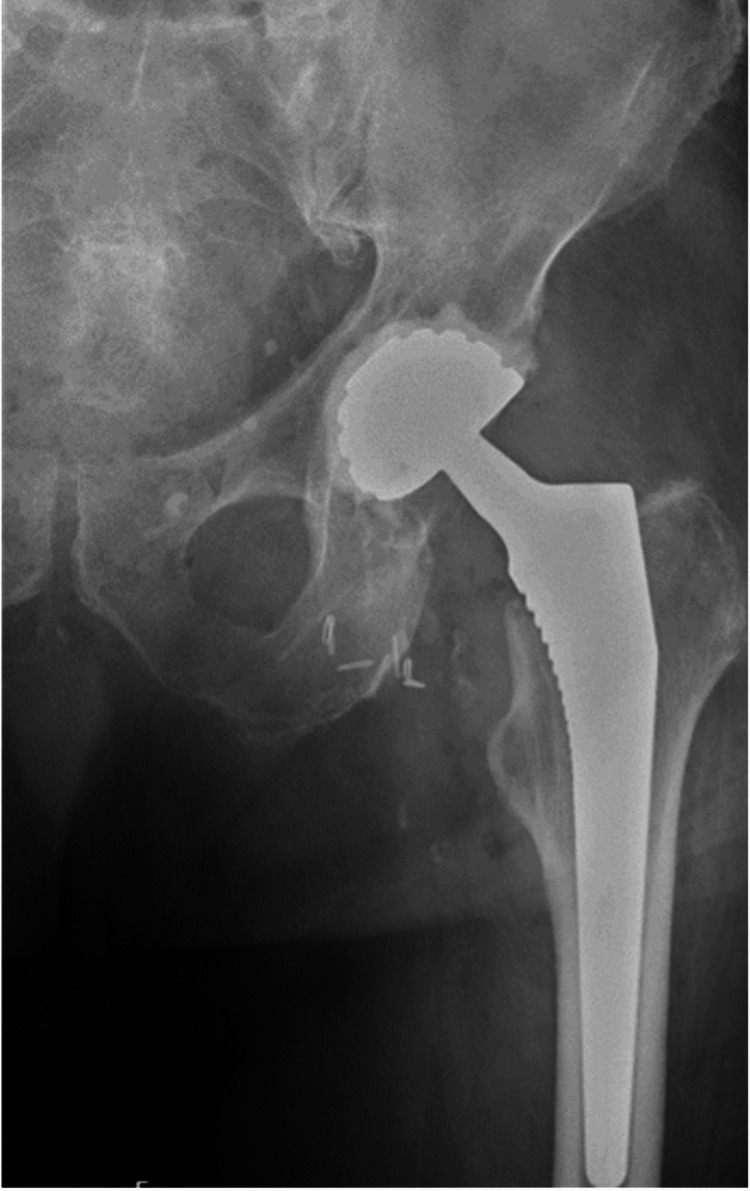
Hip radiograph after revision surgery with cemented dual-mobility acetabular component.

Acetabular component revision was performed. Intraoperatively, the PE liner was found within the gluteus medius muscle, and the femoral head was articulating directly against the metal acetabular component (Figure 4). A new cemented DM acetabular component was implanted, and the femoral stem was retained because it remained stable (Figure 5). No immediate postoperative complications were observed.

## Discussion

Early IPD occurring after attempted closed reduction of a dislocated DM THA should be regarded as a predominantly preventable, iatrogenic mode of failure. The central clinical implication is that the reduction of a dislocated DM THA must prioritize adequate anesthesia, relaxation, and the use of fluoroscopic guidance. The reduction maneuver must be gentle traction-countertraction and strict avoidance of levering maneuvers that can disengage the head-liner capture mechanism [2,4].

DM constructs reduce postoperative instability by increasing the effective head size, jump distance, and impingement-free range of motion, and they are therefore widely used in high-risk primary and revision THA [6,7]. IPD is defined as dissociation of the PE liner from the femoral head and is uncommon with contemporary designs, with recent series reporting rates as low as 0%-0.3% [8]. Although late IPD has classically been associated with long-term PE wear and progressive failure of the retentive mechanism, early IPD has been repeatedly reported in close temporal association with closed reduction attempts of a dislocated DM THA [2,4].

The mechanism is well described: during forceful manipulation, the PE liner can become trapped against the rim of the metal shell or adjacent pelvic bone while the femoral head is effectively levered, producing the so-called “bottle-opener effect” and mechanical disengagement of the head-liner interface [2,4]. Importantly, the force applied during the reduction is not an abstract variable. A major cause is the resistance of non-anesthetized (or inadequately relaxed) musculature, which increases the traction and levering required to achieve reduction and consequently increases the likelihood of liner dissociation. This consideration is directly relevant to the present case: the first reduction was performed without general anesthesia, and although immediate post-reduction radiographs appeared concentric, subtle eccentricity or early capture compromise may have been obscured. The subsequent dislocation three days later, after reduction without new major trauma, followed by eccentric femoral head positioning and CT confirmation of PE liner dissociation and migration, supports an iatrogenic mechanism temporally related to reduction attempts rather than a wear-mediated late failure process.

After any reduction of a DM THA, radiographs must be scrutinized for eccentric femoral head position within the metal shell and for soft-tissue radiolucency consistent with the migrated liner (“bubble sign”) [2,4]. When radiographic findings are subtle or equivocal, CT should be obtained promptly to confirm IPD and localize the liner [2,4]. Once IPD is identified, urgent open management is required to prevent continued metal-on-metal articulation, metallosis, and the need for more extensive reconstruction [2]. In our patient, early acetabular revision with retrieval of the migrated liner and implantation of a new cemented DM component restored stability while allowing retention of a stable femoral stem.

From a prevention standpoint, three practical steps are essential: (1) pre-procedural planning to ensure adequate anesthesia and relaxation; (2) fluoroscopy-guided reduction using controlled traction-countertraction and avoidance of levering and pivoting maneuvers at the cup rim; and (3) systematic post-reduction imaging review with a low threshold for CT if the femoral head is not perfectly concentric or the liner is not clearly accounted for [2,4]. These measures are particularly important in patients at risk of postoperative instability and in early postoperative dislocations, where muscle spasm and pain frequently drive high-force manipulations and increase the risk of iatrogenic IPD [2,4,6,7].

## Conclusions

IPD is an uncommon but potentially serious complication unique to DM THA. Although classically linked to late PE wear, early IPD can occur iatrogenically during closed reduction, particularly when performed without adequate anesthesia and muscle relaxation, which increases resistance and promotes forceful levering at the head-liner interface. This case illustrates that even correctly assembled DM constructs may dissociate when reduction maneuvers impose excessive mechanical stress on the retentive mechanism. Therefore, closed reduction of dislocated DM THA should be performed under fluoroscopic guidance with gentle traction-countertraction and is strongly recommended to be conducted under general or spinal anesthesia to minimize the risk of iatrogenic dissociation. When reduction is undertaken, clinicians must critically assess post-reduction imaging for eccentric head positioning or radiographic absence of the liner and promptly escalate to advanced imaging when suspicion persists. Ultimately, the best management of early IPD is prevention. Closed reduction of a dislocated DM THA should be performed only under adequate anesthesia and fluoroscopic guidance, avoiding forceful levering maneuvers that can precipitate liner disengagement.
